# *Per2*-Mediated Vascular Dysfunction Is Caused by the Upregulation of the Connective Tissue Growth Factor (CTGF)

**DOI:** 10.1371/journal.pone.0163367

**Published:** 2016-09-23

**Authors:** Vaishnavi Jadhav, Qianyi Luo, James M. Dominguez, Jude Al-Sabah, Brahim Chaqour, Maria B. Grant, Ashay D. Bhatwadekar

**Affiliations:** 1 Department of Ophthalmology, Eugene and Marilyn Glick Eye Institute, Indiana University School of Medicine, Indianapolis, Indiana, United States of America; 2 Department of Cell Biology, Suny Downstate Medical Center, Brooklyn, New York, United States of America; Morehouse School of Medicine, UNITED STATES

## Abstract

*Period 2*-mutant mice (*Per2*^*m/m*^*)*, which possess a circadian dysfunction, recapitulate the retinal vascular phenotype similar to diabetic retinopathy (DR). The vascular dysfunction in *Per2*^*m/m*^ is associated with an increase in connective tissue growth factor (CTGF/CCN2). At the molecular level, CTGF gene expression is dependent on the canonical Wnt/β-catenin pathway. The nuclear binding of β-catenin to a transcription factor, lymphoid enhancer binding protein (Lef)/ T-cell factor (TCF/LEF), leads to downstream activation of CTGF. For this study, we hypothesized that the silencing of *Per2* results in nuclear translocation and subsequent transactivation of the CTGF gene. To test this hypothesis, we performed immunofluorescence labeling for CTGF in retinal sections from wild-type (WT) and *Per2*^*m/m*^ mice. Human retinal endothelial cells (HRECs) were transfected with siRNA for *Per2*, and the protein expression of CTGF and β-catenin was evaluated. The TCF/LEF luciferase reporter (TOPflash) assay was performed to validate the involvement of β-catenin in the activation of CTGF. *Per2*^*m/m*^ retinas exhibited an increased CTGF immunostaining in ganglion cell layer and retinal endothelium. Silencing of *Per2* using siRNA resulted in an upregulation of CTGF and β-catenin. The TOPflash assay revealed an increase in luminescence for HRECs transfected with *Per2* siRNA. Our studies show that loss of *Per2* results in an activation of CTGF via nuclear entry of β-catenin. Our study provides novel insight into the understanding of microvascular dysfunction in *Per2*^*m/m*^ mice.

## Introduction

Circadian rhythms play an important role in regulating many biochemical, physiological, and behavioral processes such as carbohydrate metabolism, sleep-wake cycle, and feeding. Disturbed circadian rhythms or circadian dyssynchrony is observed in shift work disorders and include, jet-lag, advanced sleep phase syndrome, and irregular sleep-wake rhythm [1]. Diabetic retinopathy (DR) is a long-term complication of diabetes. During the first twenty years of diabetes, nearly all patients with Type 1 diabetes (T1D) and >60% of patients with Type 2 diabetes (T2D) will develop some form of the DR [2]. We previously identified DR as a disease of circadian dysfunction and demonstrated dampened expression of clock regulatory genes in the retina during T2D [3].

The core oscillator that drives the clock, the suprachiasmatic nucleus (SCN), is located in the hypothalamus; At the molecular level, circadian rhythms are orchestrated by interlocking autoregulatory transcription/translation feedback loops involving a set of clock genes [4]. Compelling evidence suggests that the retina is under the influence of circadian rhythms [5]. Notably, the retinal clock is independent of the SCN and can influence the rhythms generated by the SCN [6]. The clock gene rhythm in the retina is generated in a cell-autonomous manner. In the retina, melatonin release [7], dopamine synthesis [8], gamma-amino butyric acid (GABA) turnover [9] and rhodopsin gene expression [10] exhibit a physiological circadian rhythm. The retinal clock also regulates cell survival and growth processes in the eye such as photoreceptor survival in animal models of retinal degeneration [11], ganglion cell viability during aging [12] and important retinal functions such as phagocytosis, disk shedding, and corneal thickness. In mouse retina, several inner retinal cell types such as horizontal cells, bipolar cells, dopaminergic amacrine cells and ganglion cells express most of the clock regulatory genes [13].

In a previous study, we demonstrated the critical role of clock regulatory gene *Per2* and found that *Per2*^*m/m*^ mice recapitulate a vascular phenotype of DR [14]. The prominent role of the clock gene *Per2* is observed in a variety of human and mouse studies. In humans, a mutation in *Per2* leads to a condition referred to as advanced sleep phase syndrome (ASPS), which is characterized by a short sleep cycle [15, 16]. *Per2*^*m/m*^ mice possess a range of pathological disorders, such as cancer [17], shortened life-span, auto-amputations, increased alcohol consumption [18], and endothelial dysfunction [19, 20]. Therefore, understanding the mechanism of *Per2* dysregulation and the above disorders, specifically at the retina, is of intense interest to develop pharmacological targets.

CTGF is a secreted protein known to be involved in a variety of cellular events, such as angiogenesis, skeletogenesis, and wound healing [21]. In the retina, CTGF is known to be upregulated in diabetes, and its inhibition protects from the development of DR [22]. CTGF is an important downstream effector of transforming growth factor β1 (TGF-β1), a known inducer of extracellular matrix components, such as collagen and fibronectin [23]. Our previous studies on *Per2*^*m/m*^ retinas showed an increase in TGF-β1, CTGF, and fibronectin, suggesting an active involvement of the TGF-β1-CTGF pathway in vascular dysfunction of *Per2*^*m/m*^ *mice* [14]. The Wnt-β-catenin signaling pathway plays an important role in an upregulation of CTGF in the retina [22]. The nuclear binding of non-phosphorylated β-catenin to lymphoid enhancer binding protein (Lef)/ T-cell factor (TCF) TCF/LEF was demonstrated as a major regulator of CTGF activation [24].

While our previous studies on *Per2*^*m/m*^ retinas showed an increase in CTGF, the exact mechanism behind the upregulation of CTGF in *Per2*^*m/m*^ retinas remains unknown. The purpose of this mechanistic study was to determine whether silencing of *Per2* results in activation of the β-catenin-CTGF pathway and associated phenotypes. First, we performed scotopic electroretinogram (ERG) measurements and measured retinal thickness using the optical coherence tomography (OCT) in the retina of *Per2*^*m/m*^ mice. Next, we measured CTGF expression in the retina from *Per2*^*m/m*^ mice, and we assessed the effect(s) of *Per2*-silencing in HRECs on β-catenin and CTGF. Ultimately, our study highlights that the DR-like vascular phenotype observed in *Per2*^m/m^ mice is due to a β-catenin mediated increase in CTGF expression.

## Materials and Methods

### Animal Studies

All animal studies were performed in accordance with The Guiding Principles in the Care and Use of Animals (NIH) and the ARVO Statement for the Use of Animals in Ophthalmic and Vision Research. The protocol was approved by the institutional animal care and use committee of the University of Florida. The animal studies were completed while the authors were at the University of Florida, since then the research team has moved to the Indiana University School of Medicine. Wild type and *Per2*^m/m^ female mice (B6.129-Per2^tm1Drw^/J) at 12 months of age were obtained as a gift from Dr. Choogon Lee, Florida State University, Tallahassee, FL). All animal studies were performed between 11 AM and 1 PM.

For the ERG studies, the animals were dark-adapted overnight. The mice were anesthetized with a mixture of Ketamine and Xylazine. The pupils were dilated using 1% tropicamide and 2.5% phenylephrine. The platinum loop electrode (Grass Telefactor, Rhode Island, USA) was placed over the cornea after the application of Gonak (Hypromellose 2.5% solution Akorn Inc, Lake Forest, IL), and the scotopic ERG recordings were performed using an LKC NGIT-100 recording machine (LKC Technologies, Inc, Gaithersburg, MD, USA).

The OCT assessments were performed on anesthetized mice using a Bioptigen Spectral Domain Ophthalmic Imaging System (Leica Microsystems Inc, Buffalo Grove, IL). Total retinal thickness was evaluated by manually placing of calipers extending from the retinal nerve fiber layer (RNFL) up to the retinal pigment epithelium (RPE), as described previously [25]. The brightly luminous portion of the choroid under the RPE was omitted during the analysis.

### Culture of HRECs

HRECs were purchased from ATCC (Manassas, VA). Cells from passage two to seven were used for all experiments. Cells were cultured in pre-coated flasks/dishes/coverslips with attachment factor (AF), and M131 media was supplemented with microvascular growth factor (MVGS) (M131 Kit, Life Technologies, Grand Island NY) with 100U of antibiotic-antimycotic (Gibco-Life Technologies).

### Staining retinal sections for CTGF

The retinal vessels were labeled with an intravenous injection of Rhodamine-conjugated *Bandeiraea simplicifolia* (BS)-1 isolectin (0.1 mg/kg; Vector Laboratories) 30 minutes prior to euthanasia. The eyeballs from mice were enucleated, fixed in 4% paraformaldehyde and embedded in paraffin. Sections were then deparaffinized and incubated in citrate plus buffer (ScyTek laboratories, West Logan, UT) for epitope retrieval [26]. The retinal sections were then incubated with anti-CTGF primary antibodies (1:100; Abcam, Cambridge, MA) overnight at +4°C. On the following day, after careful washing of unbound primary antibodies, the retinal sections were incubated with Alexa Fluor 488 secondary antibodies (1:800; Molecular Probes-Life Technologies). Two hours after incubation with secondary antibodies, the retinal sections were washed in PBS and mounted in a SlowFade gold with DAPI mounting media and imaged using a confocal microscope (Zeiss LSM 510 META; Carl Zeiss MicroImaging GmbH, Jena, Germany).

### siRNA transfections for *Per2*

HRECs were grown and transfected with 10μM *Per2* siRNA (#s30208; Ambion-Life Technologies) using Lipofectamine RNAiMax reagent (Life Technologies) and were harvested after 24h for RNA analysis and protein analysis by Western blot. Cells transfected with negative control siRNA (#4390846, Ambion-Life Technologies) were used as a control. siRNA-transfected cells were harvested between 11 AM and 1 PM and processed for protein or mRNA expression.

### qRT-PCR for mRNA analysis

RNA was isolated using TRIzol (Life Technologies) as per the manufacturer’s protocol. One microgram of RNA was reverse transcribed using iScript cDNA synthesis kit (Bio-Rad, Hercules, CA). Gene-specific primers were used along with TaqMan Fast Universal master mix (Life Technologies), and respective mRNA levels were assessed using quantitative PCR (ViiA 7, Applied Biosystems). All genes were normalized to β-actin. Primers used were: *Per2* (Hs 00256144), CTGF (Hs 00170014), β-catenin (CTNNB1 Hs 00355049), and β-actin (Hs 01060665) from Applied Biosystems.

### Western blotting

The *Per2* siRNA-transfected cells were pelleted and lysed after 24 hrs in a RIPA buffer (R0278, Sigma-Aldrich, St. Louis, MO). Nuclear and cytoplasmic extracts were prepared using an NE-PER Nuclear and Cytoplasmic extraction kit (Thermo Fisher Scientific, Grand Island, NY) Protein concentration was estimated using the BCA assay (Pierce, Thermo Scientific, Rockford, IL), and equal amounts of proteins (50 μg) were loaded and separated on 4–12% Bis-Tris gels (Novex, Life technologies, Carlsbad, CA). Proteins were transferred to a PVDF membrane (Life Technologies), which was blocked with 5% milk or BSA as per the antibody requirement. The following antibodies were used for probing: CTGF (1:1000; Abcam), and β-catenin (1:1000; Cell Signaling Technology, Boston, MA). The PVDF membranes were incubated with the aforementioned primary antibodies overnight at +4°C. The following set of antibodies were used as a secondary loading control: α-tubulin (1:800) and Histone-H3 (1:2000; Sigma-Aldrich, St. Louis, MO). The secondary antibody incubations were performed for two hours at room temperature. The bands were visualized using ECL2 western blotting substrate (Pierce) on a XRS gel documentation system with Quantity One software (Bio-Rad, CA).

### TCF/LEF Luciferase assay

HRECs were co-transfected with a TCF/LEF construct (Cignal reporter assay kit, Qiagen Sciences, MA,) and a 10 μM *Per2* or a control siRNA as per the manufacturer’s protocol. The cells were also co-transfected with a target gene luciferase reporter constructs and constitutively expressing Renilla construct. After 24h of transfection, the cells were harvested, and lysates were prepared and measured for luminescence using a Dual Luciferase Assay system (Promega, Madison, WI) on a Synergy H1 Hybrid Reader (BioTek, Winooski, VT). The ratio of luminescence for luciferase to Renilla was plotted for all experimental conditions.

### Statistics

The data were expressed as mean ± SEM. Statistical analysis was performed using a Graph Pad-Prizm 6 (GraphPad Software La Jolla, CA) software. The statistical significance was evaluated using Student’s t-test or a Two-Way ANOVA followed by a Bonferroni’s post- hoc test. The data were considered to be statistically significant when the p-value was less than 0.05.

## Results

### *Per2*^*m/m*^ mice show decreases in ERG amplitude and retinal thickness

**Fig 1:**
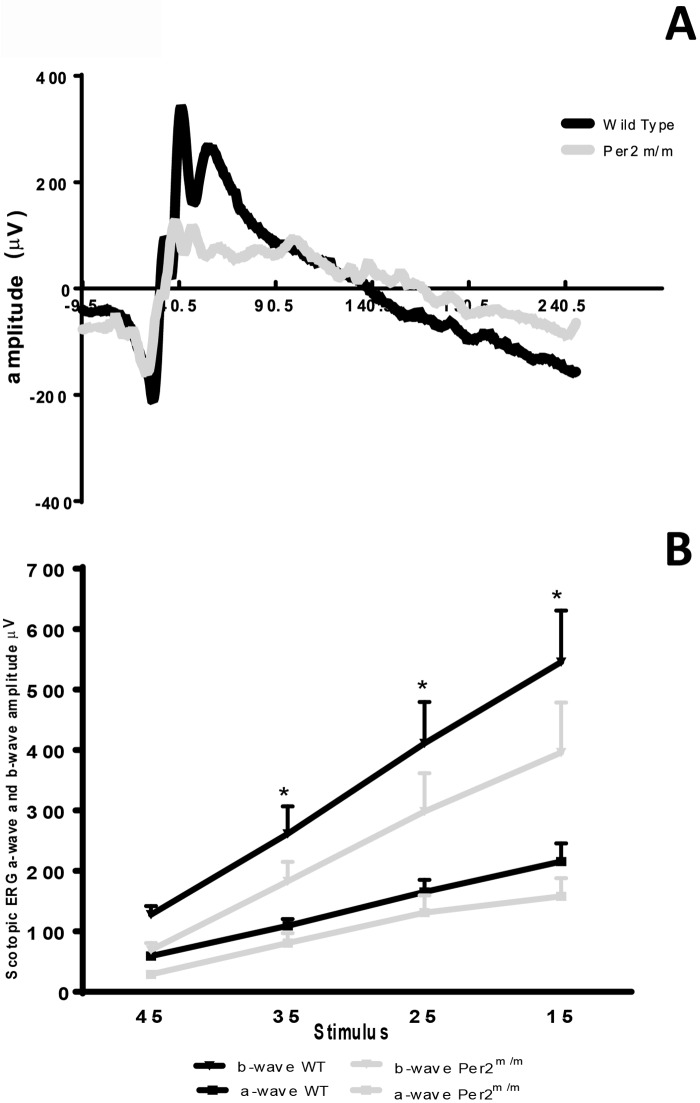
*Per2*^*m/m*^ mice exhibit decreased ERG amplitude. (A) Representative ERG trace for 15 db stimuli showing a decrease in ERG amplitude in *Per2*^*m/m*^ retinas (B) Line graph showing a similar decrease in ERG response for both 'a' and 'b' wave in *Per2*^*m/m*^ mice for a series of stimuli in the range of 45–15 db. n = 7, *p<0.05.

**Fig 2:**
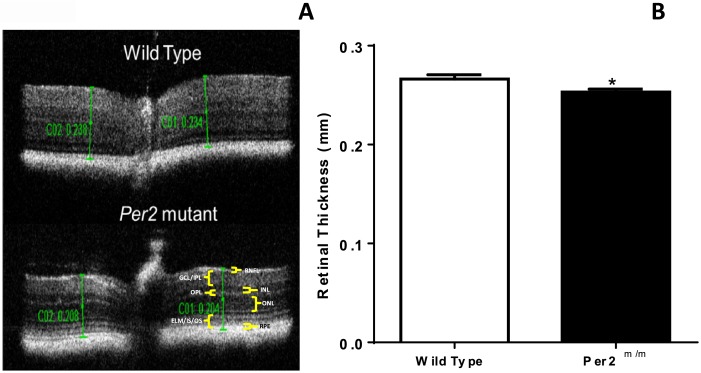
*Per2*^*m/m*^ mice show a decrease in retinal thickness. (A) The OCT scans of the whole retina showing representative images from the WT and *Per2*^*m/m*^ mice. RNFL, retinal nerve fiber layer; GCL/IPL, Ganglion cell layer/inner plexiform layer; INL, Inner nuclear layer; OPL, Outer plexiform layer; ONL, Outer nuclear layer; ELM/IS/OS, external limiting membrane/inner segment of photoreceptors/outer segment of photoreceptors; RPE, Retinal pigment epithelial layer. (B) The measurements of retinal thickness were performed by placing the 2 calipers (C01 & C02) near the optic nerve; the bar chart shows a decrease in retinal thickness of *Per2*^*m/m*^. n = 6, *p<0.05.

In order to determine the extent of neuronal dysfunction and the neurodegenerative changes in the retina of *Per2*^*m/m*^ mice, we performed scotopic ERG measurements and measured retinal thickness using the OCT. We found that the amplitude of ERG wave was reduced overall in the *Per2*^*m/m*^ retinas (Fig 1A), as both the ‘a’ wave and the ‘b’ wave were decreased in the *Per2*^*m/m*^ animals. While the difference in ERG amplitude was statistically significant for the ‘b’ wave, we observed that this difference remained insignificant for the ‘a’ wave (Fig 1B). In addition, we observed a profound decrease in retinal thickness in *Per2*^*m/m*^ mice, suggestive of neurodegenerative changes (Fig 2).

### Retinas from *Per2*^*m/m*^ mice exhibit increases in CTGF expression in retinal blood vessels

**Fig 3:**
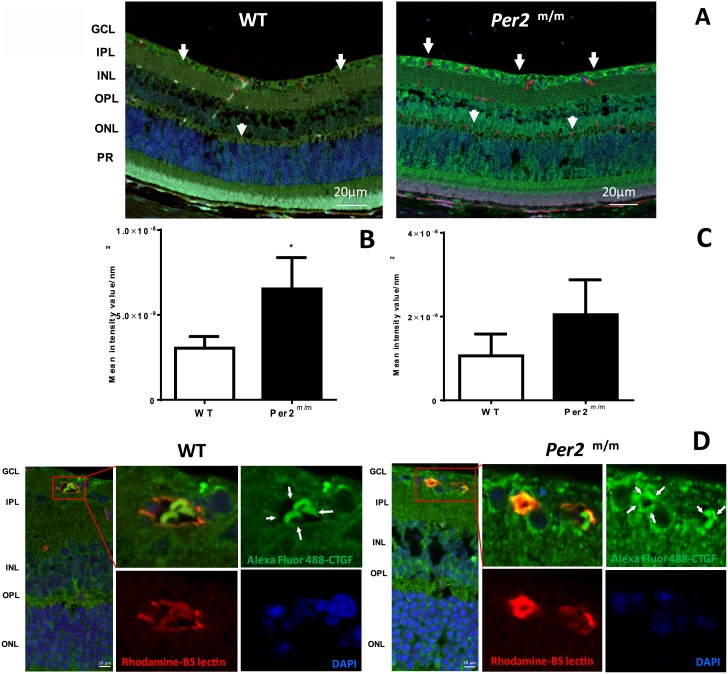
The increase in CTGF staining in *Per2*^*m/m*^ retinas. (A) Retinal sections were stained with CTGF antibodies. The photomicrographs show an increase in staining for CTGF (green) in the GCL (white arrows) and OPL (white arrows) in *Per2*^m/m^ mice; the bar-graph shows quantification of mean fluorescence intensity for (B) GCL and (C) OPL. (D) Retinal endothelial cells were stained with BS-isolectin (red) prior to euthanasia. The staining reveals an increase in CTGF expression in vessels co-stained with BS-1 isolectin. GCL, ganglion cell layer; IPL, inner plexiform layer; INL, Inner nuclear layer; OPL, outer plexiform layer; ONL, outer nuclear layer; PR, photoreceptors. n = 3.

To test whether neuronal changes of the *Per2*^*m/m*^ mice alters CTGF expression in retinal blood vessels, we stained the retinal sections with CTGF antibodies. Prior to euthanasia, the retinal vessels were stained for BS-1 isolectin with a tail vein injection of rhodamine-conjugated isolectin. The overall retinal neuropile exhibited an increase in CTGF expression (Fig 3A), which was more profound in the ganglion cell layer (GCL; Fig 3B) and the outer plexiform layer (OPL; Fig 3C). The retinal vessel wall exhibited intense staining of CTGF in *Per2*^*m/m*^ *mice* as compared to the WT animals (Fig 3D).

### siRNA *Per2* upregulates β-catenin and CTGF expression in the retina of the *Per2*^*m/m*^ mice

**Fig 4:**
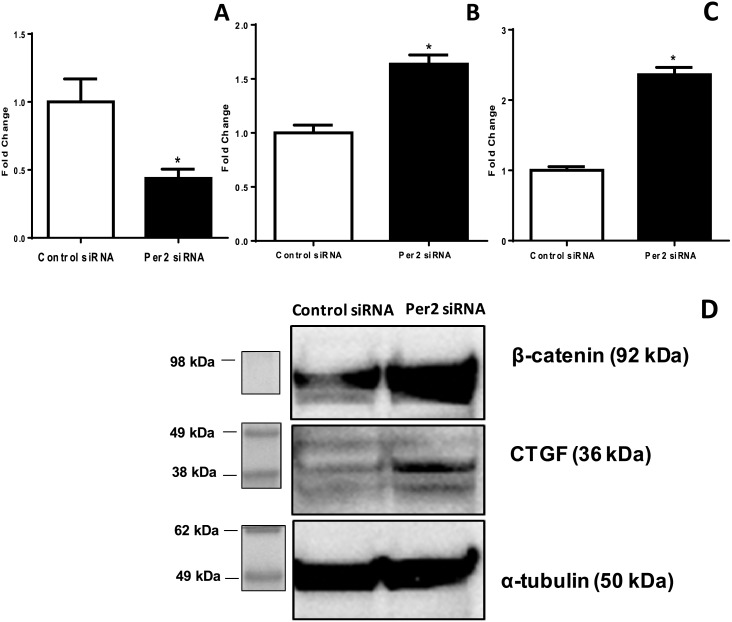
*Per2*-silencing in HRECs results in an increase in β-catenin and CTGF. HRECs were treated with either scrambled control siRNA or *Per2* siRNA; bar graphs show mRNA expression of (A) *Per2*, (B) β-catenin and (C) CTGF, n = 4. (D) Western blots show a similar change in the protein expression of β-catenin and CTGF following a treatment with siRNA *Per2*. n = 3, * p<0.05.

We used cultured human retinal endothelial cells (HRECs). The HRECs were transfected with either siRNA *Per2* or a scrambled control. The mRNA expression of *Per2*, *β-catenin* and CTGF was evaluated using qRT-PCR. As shown in Fig 4A, the *Per2* siRNA treatment resulted in a 2-fold decrease in *Per2* expression. Conversely, siRNA *Per2*-transfected HRECs showed a 1.6-fold increase in β-catenin (p<0.05; Fig 4B) and a 2.5-fold increase in CTGF (p<0.05; Fig 4C) mRNA levels. To evaluate whether changes in mRNA expression also translated to an increase in β-catenin and CTGF, we performed a western blot of HRECs transfected with siRNA *Per2*. We observed a similar increase in the protein expression of CTGF and β-catenin as a result of *Per2* silencing (Fig 4D).

### *Per2* silencing causes dislodgment and nuclear transport of β-catenin

**Fig 5:**
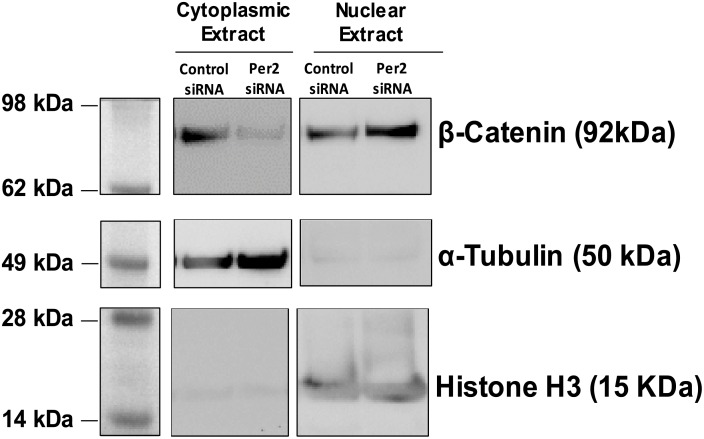
Nuclear entry of β-catenin after treatment of HRECs with siRNA *Per2*. *Per2* or control siRNA-treated HRECs were processed to separate nuclear and cytoplasmic fractions. Representative western blots showed nuclear translocation of β-catenin after treatment with *Per2* siRNA. Histone H3 and α-tubulin were used as loading controls for nuclear and cytoplasmic fractions, respectively n = 3.

To determine whether *Per2*-silencing would result in a dislodgement of β-catenin from the cytoplasm and its subsequent transfer to the nucleus, we transfected the HRECs with siRNA *Per2* and stained with CTGF antibodies. The nuclear and cytoplasmic fractions of *Per2* and control siRNA treated HRECs were separated. The Western blots were performed to determine the protein expression of β-catenin. The Histone H3 and α-tubulin were used as loading controls for nuclear and cytoplasmic fractions respectively. As shown in Fig 5, *Per2* siRNA-treated HRECs showed an increase in β-catenin staining in a nuclear fraction as compared to the cytoplasmic fraction, suggesting nuclear entry of β-catenin due to *Per2*-silencing.

### *Per2*-silencing results in nuclear binding of β-catenin resulting in activation of CTGF

**Fig 6:**
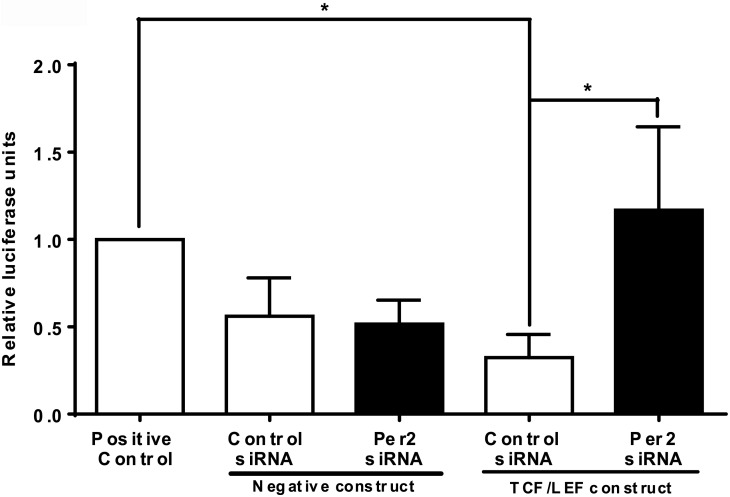
β-catenin binds to TCF/LEF factors after treatment with siRNA *Per2*. The TOPflash assay was performed to study the nuclear binding of β-catenin after treatment with siRNA *Per2*. The bar chart showing a significant increase in luciferase signal following a treatment with siRNA *Per2* when compared to a control siRNA. n = 3, * p<0.05.

We used TOPflash assay to determine whether *Per2* silencing results in a nuclear binding of TCF/LEF and downstream activation of CTGF. We co-transfected the HRECs with the *siRNA Per2* and the TOPflash plasmid. We observed a 4-fold increase (p<0.05) in luciferase activity following *Per2* silencing that was consistent with nuclear binding of TCF/LEF. (Fig 6).

## Discussion

*Per2*^*m/m*^ mice exhibit the pathologic hallmark features consistent with DR [14]. This study identified a novel mechanism of retinopathy development by defining a critical role of β-catenin-mediated CTGF increase in *Per2*^*m/m*^ mice. The *Per2*^*m/m*^ mice exhibit phenotypic defects such as a decrease in ERG amplitude and a profound decrease in retinal thickness with concurrent increase in CTGF expression. The *in vitro* studies using siRNA *Per2* reveal that β-catenin binding to TCF/LEF factors play an integral role in CTGF upregulation. Additionally, we found that the amplitude of ERG wave was reduced overall in the *Per2*^*m/m*^ retinas, suggestive of neurodegenerative changes.

TGF-β1 [27] and CTGF [28] localization in the ganglion cell layer of the retina are linked to a decrease in ERG amplitude in pathological states such as diabetes [29]. Our immunofluorescence studies provide supporting evidence of a similar increase in CTGF in ganglion cell layer of retinal sections of *Per2*^*m/m*^ mice. Therefore, we believe that a decrease in ERG amplitude in *Per2*^*m/m*^ mice is attributed, at least in part, to an activation of CTGF in retinal ganglion cells. Other retinal cell types, such as bipolar cells and Müller cells, are also known to contribute to the ERG ‘b’ wave. Thus, we speculate that there may be a similar increase in CTGF expression in these cell types; however, this requires further validation by individual cell staining. The decrease in the OCT measurements of *Per2*^*m/m*^ retinas provides further insights for undertaking mechanistic studies of the other cell types of retinal neuropile.

Our ERG and OCT findings differ from previous studies by Ait-Hymed *et al*. [30], which reported no difference in ERG amplitude and OCT recordings between WT and Per1/Per2 knockout animals. This discrepancy is potentially due to differences between the two different genetic strains. For instance, the genetic strain of *Per2*^*m/m*^ mice (*Per2*^*tm1Drw*^) used in our studies does not produce detectable protein levels of PER2 [31]. However, the strain used in studies by Ait-Hymed *et al*. (*Per2*^*tm1Brd*^*)* has the potential to generate a protein with a deletion of 87 amino acids [32]. Thus, we believe that inconsistencies in ERG and OCT findings may be attributed to translated PER2 protein.

The predominant finding of our study is defining the critical role of CTGF in the development of retinopathy in *Per2*^*m/m*^ mice. The *Per2*^*m/m*^ retinas exhibited an increased CTGF immunostaining in ganglion cell layer and retinal endothelium. We observed an upregulation of CTGF and β-catenin associated with *Per2* silencing. CTGF is known to play a critical role in the pathogenesis of DR by promoting extracellular matrix (ECM) protein production [24 25]. In this study, the animals lacking the functional CTGF allele (CTGF⁺/⁻) and made diabetic using streptozotocin (STZ) did not develop classical symptoms of DR, such as an increase in ECM thickening, pericyte dropout and an increase in acellular capillaries. In contrast, the animals that possessed the (CTGF^+/+^) functional allele developed a full spectrum of the above features of DR. Taken together, this suggests that CTGF is involved in the pathological change of the retina in diabetes. Our study further ascertained an important role of CTGF in retinal vascular dysfunction due to lack of *Per2*.

Our studies show that clock function (i.e., Per2), β-catenin and CTGF are mechanistically interlinked, and this trio together might represent an important determinant in the pathogenesis of DR. For instance, previous studies suggest that hyperactivity of β-catenin is linked to pathological retinal neovascularization [33] and hereditary vascular disorders [34, 35]; and inhibition of Wnt pathway has been shown to be protective in DR due to downregulation of the CTGF [22]. Our study demonstrated that *Per2* is an important upstream regulator of the β-catenin-CTGF pathway in DR, and maintaining optimum levels of *Per2* are necessary for protection from DR.

We previously reported a loss of junctional integrity of VE-Cadherin in *Per2*^*m/m*^ mice [14]. β-catenin is a critical junctional protein. The previous study suggests an important role of CTGF in proliferative DR [36]. However, the involvement of CTGF in retinal permeability is not well-defined. We believe that β-catenin is critical in mediating an increase in CTGF expression in *Per2*^*m/m*^ mice. Our studies using a TOPflash assay reveal that the silencing of *Per2* results in a translocation of β-catenin from the cytoplasm to the nucleus and subsequent downstream activation of CTGF. These findings indicate a β-catenin-mediated increase in CTGF expression is responsible for the DR-like vascular phenotype observed in *Per2*^*m/m*^ mice. Moreover, the β-catenin expression is increased following *Per2*-silencing in HRECs, in agreement with previous studies performed on colon carcinoma cells [17]. Our study demonstrated a similar increase in β-catenin in both healthy cells and the retina.

Another possible mechanism of nuclear translocation of β-catenin may be the result of the common phosphorylation and degradative pathway shared by both PER2 protein and β-catenin. The casein kinase 1 (CK1) acts as a key mediator that regulates nuclear import of β-catenin by virtue of its dual control over the phosphorylation of the PER2 and β-catenin. We speculate that in the *Per2*^*m/m*^ mice, CK1 shifts its enzymatic activity towards PER2, leading to its enhanced degradation and, therefore, reduced β-catenin phosphorylation. This, in turn, results in the nuclear entry of β-catenin, leading to downstream activation of targets such as CTGF.

In conclusion, our study identified a novel pathway of retinopathy progression in *Per2*^*m/m*^ mice by ascertaining the critical role of β-catenin-mediated CTGF increase. Further, our study paves a way for the development of pharmacological targets that, in the future, may curtail the development of DR.
